# Association between advanced lung cancer inflammation index and all-cause and cardiovascular mortality among stroke patients: NHANES, 1999–2018

**DOI:** 10.3389/fpubh.2024.1370322

**Published:** 2024-04-18

**Authors:** Xiaokun Chen, Chunzhan Hong, Zeming Guo, Hongyu Huang, Lichao Ye

**Affiliations:** Department of Neurology, The Second Affiliated Hospital, Fujian Medical University, Quanzhou, China

**Keywords:** stroke, advanced lung cancer inflammation index, National Health and Nutrition Examination Survey, all-cause mortality, CVD mortality

## Abstract

**Background:**

Stroke was a major global public health challenge, and its prognosis was remarkably associated with inflammation levels and nutritional status. The advanced lung cancer inflammation index (ALI) was a comprehensive indicator that combined inflammation and nutritional status. Currently, the relationship between ALI and the prognosis of stroke patients was not yet known. The purpose of the current study was to estimate their relationship.

**Methods:**

Cohort data from the National Health and Nutrition Examination Survey (NHANES) 1999–2018 were collected. The association between ALI and all-cause and cardiovascular disease (CVD) mortality in stroke patients was estimated using a multivariable adjusted Cox model. Their non-linear relationship was analyzed by restricted cubic spline analysis. Sensitivity analysis was constructed through stratified analysis and interaction analysis.

**Results:**

1,440 stroke patients were included in this study. An elevated ALI was significantly related to a reduced risk of all-cause mortality in stroke patients but not related to CVD mortality. A reverse J-shaped non-linear association between ALI and all-cause mortality in stroke patients, with an inflection point at 83.76 (the lowest of the mortality risk). On the left side of the inflection point, for each 10 U increase in ALI, there was a 16% reduction in the risk of all-cause mortality. However, on the right side, the risk increased by 6%. There was no remarkable interaction between stratified variables and ALI.

**Conclusion:**

This was the first study on the relationship between ALI and all-cause and CVD mortality in stroke patients. Elevated ALI was closely associated with a reduced risk of all-cause mortality. A reverse J-shaped non-linear relationship existed between the two, with an inflection point at 83.76. These findings implied that controlling the ALI of stroke patients within an appropriate range was crucial for their prognosis (such as weight management, albumin supplementation, anti-inflammatory treatment). The dynamic variation in ALI was also advantageous for clinicians in establishing personalized ALI criteria to maximize the long-term survival of stroke patients.

## Introduction

Presently, stroke remains the second leading cause of death worldwide, second only to ischemic heart disease. There are around 100 million stroke cases worldwide, with a stroke incidence rate of 5% (1). Despite advancements in stroke treatment, age-standardized death rates were 96.4 per 100,000 in males and 73.5 per 100,000 in females (1). Stroke was an acute cerebrovascular disease that could lead to speech, cognitive, and motor impairments, and even death (2). Stroke represented a major global public health challenge, and researchers have been striving to improve its prognosis by addressing its risk factors.

The etiology and pathological progression of stroke were complex, with studies indicating that inflammation was involved in the occurrence and development of stroke (3). Elevated levels of inflammation were associated with increased stroke incidence and mortality risk. In stroke patients, markers like interleukin-6 (IL-6), tumor necrosis factor (TNF), and neutrophil counts were markedly increased (4–6). Hence, stroke was regarded as an inflammatory disease. Prolonged chronic inflammation could result in insulin resistance via inflammatory factors like TNF and C-reactive protein (CRP), subsequently causing a decrease in body weight and albumin levels (7–9). Prior research has shown that nutritional status indicators, including albumin and body mass index (BMI), were closely associated with the prognosis of stroke patients (10, 11). Consequently, inflammation could affect the prognosis of stroke patients not only directly but also indirectly through albumin and BMI. Hence, assessing the prognosis of stroke patients solely based on inflammation markers might not be comprehensive, and there was an urgent requirement for new predictive indicators to holistically evaluate the prognosis of stroke patients.

The advanced lung cancer inflammation index (ALI) was a novel comprehensive index that integrated inflammation and nutritional status, encompassing parameters such as albumin, BMI, and neutrophil to lymphocyte ratio (NLR). Originally, ALI was utilized to evaluate the prognosis of lung cancer patients (12), and later, it was expanded for applications in colorectal, and pancreatic cancers (13, 14). Because ALI could evaluate both inflammation and nutritional status concurrently, it has attracted attention for its role in inflammation-related conditions like heart failure, coronary artery disease, hypertension, and diabetes (15–18). Yet, the relationship between ALI and the long-term survival of stroke patients was currently not well understood.

The National Health and Nutrition Examination Survey (NHANES) was a nationally representative, multi-stage, large-sample study, authorized and constructed by the National Center for Health Statistics (NCHS), primarily encompassing nutritional and health data of the American population. The detailed description of NHANES has been published elsewhere (19).

The objective of the current study was to evaluate the association between ALI levels and the risk of all-cause and cardiovascular disease (CVD) mortality in stroke patients. To facilitate management in a clinical setting, we further quantified the impact of dynamic changes in ALI on the prognosis of stroke patients.

## Methods

### Study population

**Figure 1:**
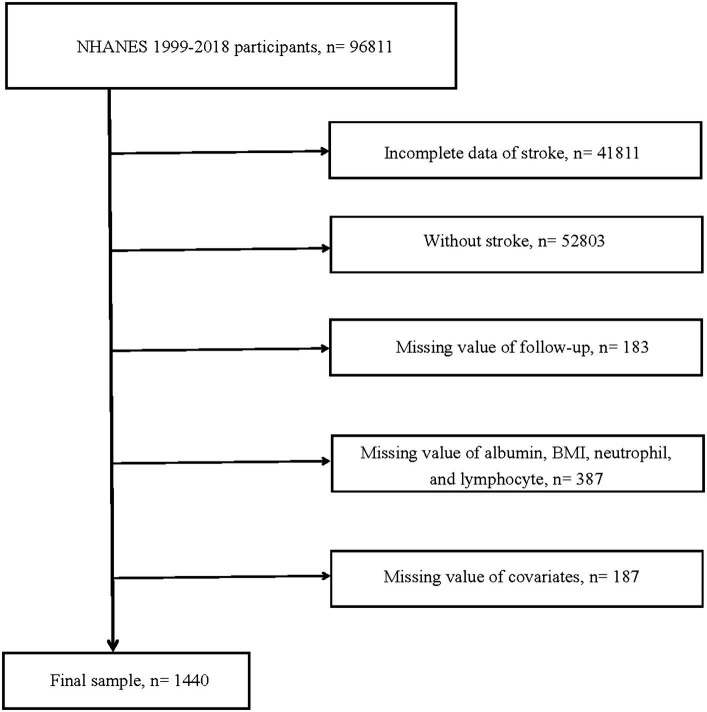
Flow chart of study participants. BMI, body mass index.

Figure 1 presented the detailed process of this research. Information of 96,811 participants from 1999 to 2018 was sourced from the NHANES. 41,811 participants with incomplete stroke data were excluded. 52,803 participants without stroke. 183 participants lacking data on follow-up time and survival status. 387 participants missing information on albumin, BMI, neutrophils, and lymphocytes. 187 individuals without information on covariates. Stroke was determined based on the results of the standard questionnaire. Specifically, when participants answered “yes” to question “Ever told you had a stroke,” they were considered to have had a stroke.

### Determination of ALI

ALI consisted of albumin, BMI, and NLR, defined as albumin*BMI/NLR (neutrophil counts divided by lymphocyte counts). Patients with stroke were categorized into three groups based on their ALI levels: Quantile1 group, Quantile2 group, and Quantile3 group.

### Mortality

All-cause and CVD mortality were determined based on data released by the National Center for Health Statistics. The cutoff date was December 31, 2019. The definition of CVD mortality was based on ICD-10 codes (I00–I078) (20).

### Covariates definitions

The following covariates were extracted: age, gender, race, BMI, smoking habits, alcohol consumption, a history of hypertension and diabetes, albumin, neutrophil counts, lymphocyte counts, uric acid, total cholesterol, and lactate dehydrogenase (LDH). The missing data for these covariates were excluded from the current study. The detailed definition and classification of the above covariates were shown in Supplementary material.

### Statistical analysis

Statistical analyses were carried out using R software (version 4.3.1), with a *p*-value of less than 0.05 deemed statistically significant. The statistical analyses of the current study were conducted strictly according to the recommendations of NHANES, taking into account sample weights, clustering, and stratified analysis. Wilcoxon rank-sum test was used for continuous variables, and chi-squared test was used for categorical variables. Kaplan–Meier analysis was conducted to preliminarily identify the association between ALI levels and all-cause, CVD mortality in stroke patients. Subsequently, multivariate Cox analysis was utilized to further explore the reliability of the aforementioned relationship after adjusting for multiple covariates. This study presented findings from four different models. The Crude model referred to the unadjusted model. Model 1 considered demographic factors such as age, gender, and race. Model 2 incorporated demographic factors, smoking habits, alcohol intake, and a history of hypertension and diabetes. Apart from the covariates in Model 2, Model 3 further included uric acid, total cholesterol, and LDH. This model was of paramount significance and was the primary focus of this study. To clarify the non-linear relationship between ALI levels and all-cause and CVD mortality in stroke patients, we employed restricted cubic spline (RCS) analysis. Once a non-linear relationship was identified, a recursive algorithm was applied to determine its inflection point. A segmented Cox regression model was conducted to further estimate the threshold effect. Furthermore, we also assessed the impact on prognosis of stroke for every 10 U change in ALI, enabling clinicians to dynamically evaluate the implications. The robustness of this study was enhanced through sensitivity analysis. For instance, we constructed stratified analyses combined with interaction analyses to jointly assess potential interactions between ALI levels and stratified variables.

## Results

### Baseline characteristics

A total of 96,811 individuals were extracted from NHANES, and after applying exclusion criteria, ultimately, this study encompassed 1,440 stroke patients, with an average age of 64.22 years and a mean ALI value of 63.35. Compared to group Quantile 1, groups Quantile 2 and Quantile 3 demonstrated different baseline characteristics. Their average values of ALI, BMI, albumin, lymphocyte counts, total cholesterol were higher, and the female ratio were elevated. However, the average age, neutrophil count, NLR were lower, and the proportion of White or diabetic patients were lower. Regarding uric acid, LDH, smoking and drinking status, and a history of hypertension, no differences were observed between the three groups. Detailed results were displayed in Table 1.

**Table 1:** Baseline demographic and medical characteristics of patients with stroke in the NHANES 1999–2018 cohort.

Characteristics	ALI				*p*-value
	Total	Quantile 1 32.67 [2.89, 44.11]	Quantile 2 54.69 (44.11, 68.18]	Quantile 3 88.94 (68.18, 893.63]	
Participants, *n*	1,440	480	480	480	
ALI, mean	63.35 (60.48, 66.22)	30.78 (29.71, 31.85)	54.74 (54.02, 55.46)	102.41 (96.32, 108.50)	< 0.0001
Age, year	64.22 (63.14, 65.31)	67.81 (66.10, 69.51)	63.29 (61.38, 65.20)	61.90 (60.44, 63.37)	< 0.0001
Gender, *n* (%)					0.01
Female	707(56.45)	195 (48.92)	243 (58.53)	269 (61.22)	
Male	733 (43.55)	285 (51.08)	237 (41.47)	211 (38.78)	
Race, *n* (%)					< 0.0001
White	778 (73.88)	301 (79.12)	279 (77.30)	198 (65.46)	
Non-White	662 (26.12)	179 (20.88)	201 (22.70)	282 (34.54)	
BMI, Kg/m^2^	29.90 (29.43, 30.38)	26.83 (26.15, 27.51)	29.78 (29.11, 30.45)	32.87 (32.00, 33.74)	< 0.0001
Albumin, g/dL	4.15 (4.13, 4.17)	4.09 (4.05, 4.12)	4.15 (4.11, 4.20)	4.20 (4.16, 4.23)	< 0.001
Neutrophil, K/uL	4.55 (4.45, 4.66)	5.45 (5.26, 5.65)	4.65 (4.51, 4.79)	3.62 (3.48, 3.77)	< 0.0001
Lymphocyte, K/uL	2.10 (2.01, 2.19)	1.50 (1.44, 1.57)	2.08 (2.01, 2.15)	2.67 (2.42, 2.92)	< 0.0001
NLR, mean	2.54 (2.44, 2.64)	3.98 (3.75, 4.21)	2.28 (2.22, 2.34)	1.50 (1.44, 1.55)	< 0.0001
Uric acid, umol/L	344.59 (338.08, 351.11)	342.45 (331.54, 353.36)	342.24 (330.71, 353.76)	349.05 (338.23, 359.87)	0.6
Total cholesterol, mmol/L	4.95 (4.87, 5.04)	4.75 (4.61, 4.89)	4.93 (4.82, 5.04)	5.16 (4.99, 5.32)	0.002
Lactic dehydrogenase, u/L	1.33 (1.30, 1.36)	1.35 (1.31, 1.40)	1.31 (1.26, 1.36)	1.32 (1.26, 1.38)	0.52
Smoke status, *n* (%)					0.85
No/Former	1,110 (75.47)	372 (74.33)	364 (75.62)	374 (76.35)	
Yes	330 (24.53)	108 (25.67)	116 (24.38)	106 (23.65)	
Alcohol, *n* (%)					0.33
No/Former	767 (49.16)	256 (48.22)	263 (48.23)	248 (51.01)	
Mild to moderate	538 (40.88)	192 (44.35)	159 (39.57)	187 (39.06)	
Yes	135(9.96)	32(7.43)	58 (12.20)	45(9.93)	
Hypertension, *n* (%)					0.37
No	269 (21.56)	84 (18.43)	91 (23.05)	94 (22.88)	
Yes	1,171 (78.44)	396 (81.57)	389 (76.95)	386 (77.12)	
Diabetes, *n* (%)					0.01
No	879 (65.18)	320 (71.77)	286 (64.59)	273 (59.74)	
Yes	561 (34.82)	160 (28.23)	194 (35.41)	207 (40.26)	

ALI, advanced lung cancer inflammation index; BMI, body mass index; NLR, neutrophil to Lymphocyte ratio.

Values are weighted mean (IQR) for continuous variables or numbers (weighted %) for categorical variables. Wilcoxon rank-sum test was used for continuous variables, and chi-squared test with Rao & Scott’s second-order correction was used for categorical variables.

### Kaplan–Meier analysis

**Figure 2:**
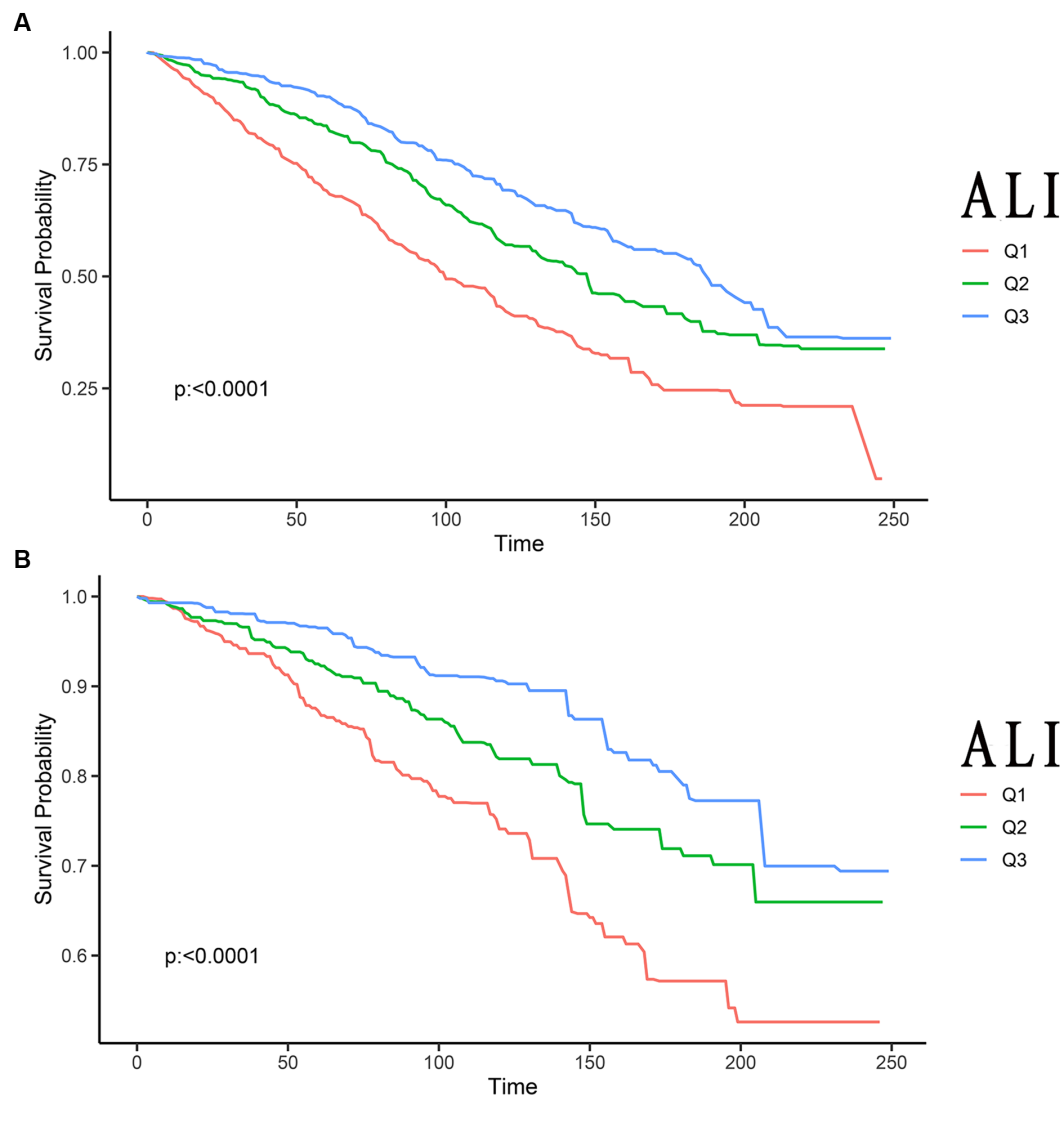
Kaplan–Meier survival curves of ALI impact on long-term all-cause **(A)**, and CVD **(B)** mortality in patients with stroke (weighted). ALI, advanced lung cancer inflammation index; CVD, cardiovascular disease; Q1, Quantile 1; Q2, Quantile 2; Q3, Quantile 3.

The Kaplan–Meier analysis served to initially distinguish the relationship between all-cause mortality and CVD mortality among ALI and stroke patients. Among the 1,440 stroke patients, 677 deaths from all causes were recorded, and 206 deaths were attributable to CVD. The Kaplan–Meier curves depicting ALI levels in relation to all-cause and CVD mortality among stroke patients were illustrated in Figure 2, with results indicating that an elevated ALI level correlated with a decreased risk of both all-cause and CVD mortality (*p* < 0.0001, *p* < 0.0001, respectively).

### Multivariate cox analysis

Table 2 displayed the results of four Cox models. Owing to Model3 including a more comprehensive set of covariates, it better mitigated the impact of confounders. Hence, the outcomes of Model3 were pivotal, and they constituted the primary emphasis of this study. The results of Model3 suggested that the higher the ALI level, the lower the risk of all-cause mortality in stroke patients. Compared with the Quantile 1 group, the Hazard Ratio (HR) for Quantile 2 and Quantile 3 groups were 0.71 (0.57, 0.89) and 0.52 (0.41, 0.66) (*p*_trend_ = 0.01), respectively. Yet, this trend was not found in CVD mortality. Compared to the reference group, the HR for CVD mortality in Quantile 2 and Quantile 3 groups were 0.74 (0.52, 1.04) and 0.47 (0.32, 0.70) (*p*_trend_ = 0.13), respectively. The findings of Model 1 and Model 2 were consistent with the Model3. Although the trend in CVD mortality reduction from the Quantile 1 group to the Quantile 2 and Quantile 3 groups was not statistically significant, we found that, when compared solely with the Quantile 1 group, the risk of CVD mortality in the Quantile 3 group remarkably decreased by 48% (0.52 [0.41, 0.66]).

**Table 2:** Relationships of ALI with all-cause, and CVD mortality in patients with stroke from the NHANES 1999–2018 cohort.

	Crude	Model 1	Model 2	Model 3
	HR, 95% CI	HR, 95% CI	HR, 95% CI	HR, 95% CI
*All-cause mortality*				
Quantile 1	ref	ref	ref	ref
Quantile 2	0.60 (0.46, 0.79)	0.78 (0.62, 0.97)	0.73 (0.59, 0.91)	0.71 (0.57, 0.89)
Quantile 3	0.44 (0.34, 0.56)	0.59 (0.46, 0.74)	0.56 (0.45, 0.71)	0.52 (0.41, 0.66)
Per 10 U increment	0.93 (0.88, 0.99)	0.97 (0.92, 1.02)	0.97 (0.92, 1.02)	0.96 (0.91, 1.02)
*p for trend*	<0.001	0.03	0.01	0.01
*CVD mortality*				
Quantile 1	ref	ref	ref	ref
Quantile 2	0.61 (0.43, 0.87)	0.80 (0.57, 1.13)	0.76 (0.54, 1.08)	0.74 (0.52, 1.04)
Quantile 3	0.39 (0.25, 0.59)	0.53 (0.35, 0.81)	0.52 (0.35, 0.78)	0.47 (0.32, 0.70)
Per 10 U increment	0.89 (0.84, 0.95)	0.93 (0.88, 0.99)	0.93 (0.88, 0.98)	0.91 (0.86, 0.97)
*p for trend*	0.01	0.23	0.17	0.13

ref, reference; HR, hazard ratios; CI, confidence interval; CVD, cardiovascular disease; LDH, lactic dehydrogenase.

Values are n or weighted HR (95% CI). Model 1: adjusted for age (years), gender (male or female), and race or ethnicity (White, Non-White). Model 2: model 1+ adjusted for smoke status (no/former or yes), alcohol (no/former, mild to moderate or heavy), hypertension (no or yes), and diabetes (no or yes). Model 3: model 2+ adjusted for uric acid (umol/L), total cholesterol (mmol/L), and LDH (u/L).

Notably, the impact of dynamic changes in ALI on prognosis was also of great concern. For each 10 U variation in ALI, the HRs for all-cause and CVD mortality were 0.96 (0.91, 1.02) and 0.91 (0.86, 0.97), respectively.

### Non-linear relationships

**Figure 3:**
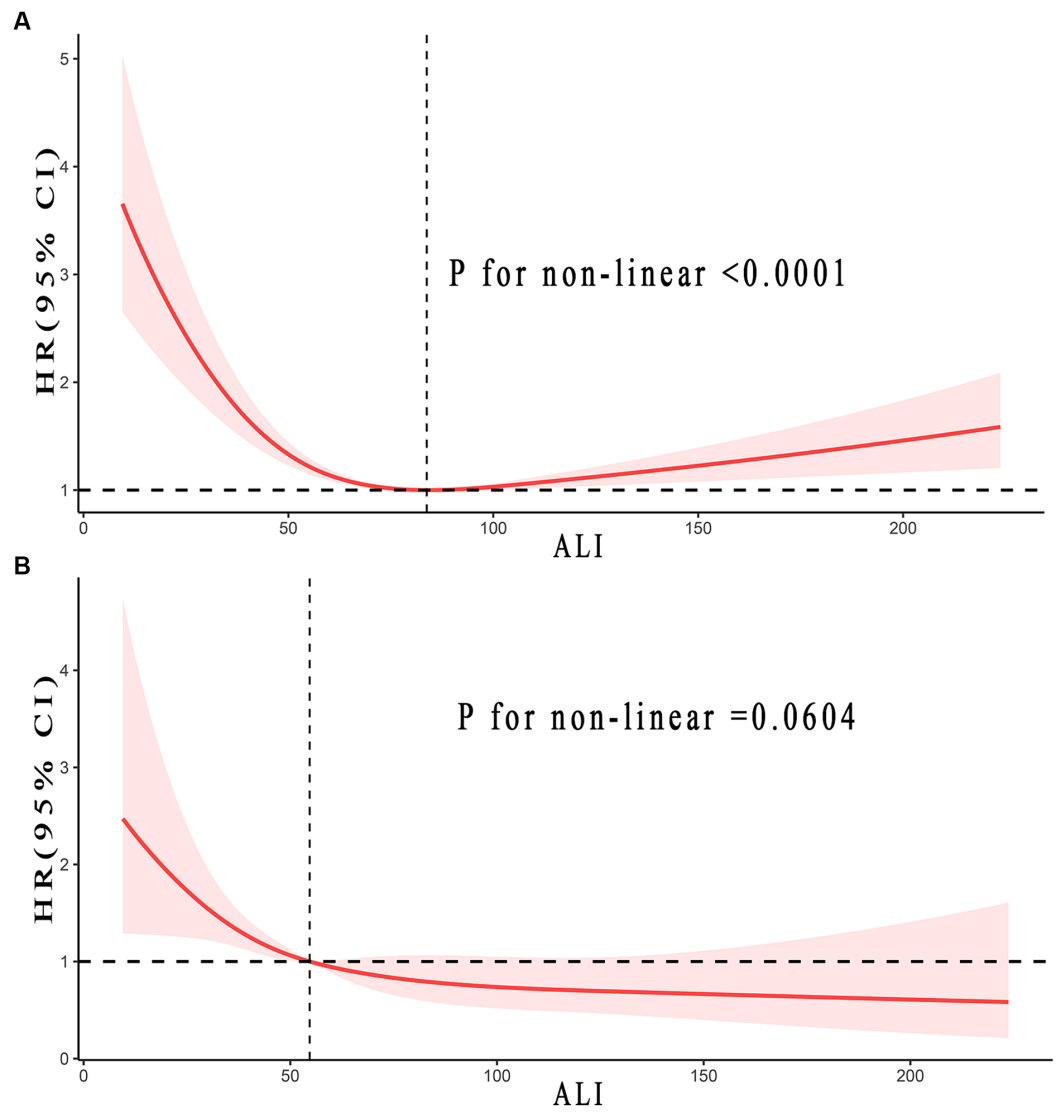
Relationship between ALI and all-cause **(A)**, and CVD **(B)** mortality in patients with stroke. Adjusted for age, gender, race, smoke status, alcohol, a history of hypertension and diabetes, uric acid (umol/L), total cholesterol (mmol/L), and LDH (u/L). The solid and red shadow represent the estimated values and their 95% CIs, respectively. ALI, advanced lung cancer inflammation index; CVD, cardiovascular disease; LDH, lactic dehydrogenase.

Figure 3 illustrated the non-linear relationship between ALI levels and the long-term prognosis of stroke patients. In terms of all-cause mortality in stroke patients, an inverse J-shaped non-linear relationship was found (*p* < 0.0001). Yet, this trend was not observed in CVD mortality (*p* = 0.0604).

The study identified inflection points was 83.76 concerning all-cause mortality in stroke patients. Every 10 U increase in ALI indicated a 16% reduction in the risk of all-cause mortality when ALI was less than 83.76 (0.84, 0.79–0.90, *p*_trend_ < 0.0001). Yet, when ALI was greater than 83.76, it implied a 6% increased risk in all-cause mortality (1.06, 1.04–1.08, *p*_trend_ < 0.0001). The results implied that with the rise in ALI, the all-cause mortality risk for stroke patients initially decreased before increasing. Conversely, CVD mortality seemed to follow a consistently downward trend. Detailed outcomes were presented in Figure 3 and Table 3.

**Table 3:** Threshold effect analysis of ALI on all-cause mortality in patients with stroke from the NHANES 1999–2018 cohort.

	All-cause mortality	
	Per 10 U increment	*p*-value
<83.76	0.84 (0.79, 0.90)	<0.0001
>83.76	1.06 (1.04, 1.08)	<0.0001

ALI, advanced lung cancer inflammation index; LDH, lactic dehydrogenase.

Values are n or weighted HR (95% CI). Model is adjusted for age (years), gender (male or female), race or ethnicity (White, Non-White), smoke status (no/former or yes), alcohol (no/former, mild to moderate or heavy), hypertension (no or yes), diabetes (no or yes), uric acid (umol/L), total cholesterol (mmol/L), and LDH (u/L).

### Sensitivity analysis

Sensitivity analysis was conducted through stratified analysis and interaction analysis. Stratified analyses for all-cause and CVD mortality among stroke patients, aiming to assess potential interactions between stratified variables and ALI, were depicted in Supplementary Tables S1, S2. There were no evident interactions observed in either all-cause or CVD mortality. Furthermore, Supplementary Table S3 illustrated the stratified analysis of all-cause mortality in stroke patients, categorized by the inflection points, assessing possible interactions between stratified variables and ALI. No notable interactions were detected. Given that no covariates in this study have an interaction effect with ALI, this implies that the findings were dependable, consistent, and have a wide clinical applicability.

## Discussion

This was the first study exploring the relationship between ALI levels with all-cause and CVD mortality among stroke patients, founded on a comprehensive and publicly accessible representative cohort. The Cox model adjusted for multiple factors demonstrated that a rise in ALI evidently correlated with a decrease in the risk of all-cause mortality for stroke patients, but it was unrelated to CVD mortality. According to RCS analysis, the all-cause mortality risk was at its lowest when ALI was at 83.76. An inverse J-shaped non-linear association was founded between them. Using the inflection point as the boundary, ALI on the left side has a protective effect. An increase of 10 U in ALI reduced the all-cause mortality risk by 16%. On the right side, the risk increases by 6%. Sensitivity analysis displayed no significant interaction between stratified variables and ALI.

Globally, stroke presented a major public health concern, ranking as the second primary cause of death following ischemic heart disease. Historically, stroke has been considered an inflammatory disease. Chronic low-grade inflammation could lead to atherosclerosis, hypertension, diabetes, and obesity, all of which were risk factors for stroke (7). Post-stroke, damaged or dead nerve cells released damage-associated molecular pattern factors, leading to localized inflammatory responses at the lesion site (3). Numerous studies have shown a remarkable elevation in IL-6, TNF, CRP, and neutrophils in patients with stroke. Higher levels of inflammation correlated with poorer outcomes for these individuals (4–6). Such evidence pointed to inflammation being both a precursor and a consequence of stroke. Importantly, inflammation could result in malnutrition, evident by a reduction in albumin and weight loss. A close relationship existed between nutritional status and the prognosis for stroke patients (7–11, 21). On one hand, low albumin levels were not only associated with an increased risk of stroke but also with worse outcomes. On the other hand, an elevated BMI could lead to an increased risk of stroke and mortality. Consequently, we contended that a simultaneous consideration of inflammation and nutritional status was essential for an accurate and holistic evaluation of stroke patient prognosis.

ALI, composed of BMI * albumin/NLR, serves as a composite index integrating inflammation and nutritional status. Initially, ALI was applied in lung cancer research and later expanded to colorectal, pancreatic cancer, and some inflammation-related diseases such as hypertension and diabetes (12–15, 18). To date, no studies have evaluated the relationship between ALI and all-cause and CVD mortality of stroke patients. In our research, we initially identified that an elevated ALI remarkably correlated with a decrease in all-cause mortality among stroke patients, without association with CVD mortality.

An inverse J-shaped non-linear relationship was first identified between ALI and all-cause mortality in stroke patients. Several elements might underlie this complex relationship. First and foremost, elevated neutrophil counts in prior studies implied non-specific inflammation, whereas decreased lymphocyte levels pointed towards compromised immunity. Consequently, NLR represented the immunological status and inflammatory reaction of an individual (22). The average NLR level in this study was 2.54, higher than the average levels in previously studied healthy individuals (1.65–2.11) (23, 24). Previous study indicated activated leukocytes released reactive oxygen species through neutrophils and cytokines, hence, promoting systemic inflammation and endothelial damage (25). Furthermore, a correlation between elevated NLR and poorer prognoses in stroke patients was found in prior studies (26–29). The findings in Tables 1, 2 revealed that, spanning from group Quantile1 to Quantile3, there was a pronounced decrease in NLR, paralleled by a substantial decline in the risk of all-cause mortality. Thus, we posited a consistent trend linking NLR with all-cause mortality risk in stroke patients: a decline in NLR correlated with a concurrent reduction in mortality risk.

Secondly, serum albumin was a frequently utilized marker for assessing nutritional status. Previous research indicated that higher albumin levels correlated with lower incidence of stroke and fewer complications caused by stroke (such as speech, cognitive, and motor impairments) (30). Furthermore, owing to its anti-inflammatory effects, albumin served an essential role in stroke therapy. Stroke patients with higher albumin levels have a better prognosis compared to those with lower levels (30). This evidence pointed to a strong link between albumin levels, the onset of stroke, progression of complications, and the outcome. The findings from Tables 1, 2 indicated a remarkable rise in albumin levels from group Quantile1 to Quantile3, with a corresponding notable decrease in the risk of all-cause mortality. Hence, we posited that there was an inverse relationship between albumin levels and the risk of all-cause mortality in stroke patients. That was, as albumin increased, the risk of death decreased correspondingly.

Additionally, BMI was another frequently used indicator for assessing nutritional status. Obesity was defined as having a BMI of ≥30. Obesity was often a high-risk factor for various diseases, including stroke (31). Notably, the relationship between BMI and the prognosis of stroke patients was controversial. Prior study indicated that stroke patients with higher BMI tend to have reduced mortality. This phenomenon was also referred to as the ‘obesity paradox’ in stroke, meaning that obesity correlated with a lower mortality after stroke (32). A possible reason could be that stroke resulted in a stressed state in the body and reduced energy intake, and obese individuals have more energy reserved. On the other hand, patients with a high BMI possessed stronger anti-inflammatory capabilities, and inflammation was closely related to stroke prognosis (33–36). However, several studies suggested that the relationship between BMI and stroke prognosis was not simply linear. A meta-analysis involving 95,651 patients found a significant inverse J-shaped non-linear relationship between BMI and the all-cause mortality from strokes. When BMI was below 25, it has a protective effect against all-cause mortality (36). Another research similarly pointed to a reverse J-shaped non-linear relationship between BMI and all-cause death from strokes. The lowest risk of mortality was observed when the BMI was 23.07 (37). Furthermore, chronic obesity could induce the production of ROS in the myocardium, while myocardial cells protected the myocardium from stress damage by enhancing antioxidant responses (38). Research displayed that homocysteine was not only associated with obesity but also correlated with ROS concentration. Homocysteine might play a significant role in this context (39, 40). Based on this evidence, we contended that BMI played a significant role in the inverse J-shaped relationship between ALI and all-cause mortality in stroke patients.

Abundant studies indicated that inflammation was involved throughout the entire process of atherosclerosis occurrence, development, and related cardiovascular events (41). Elevated levels of CRP, as one of the inflammation biomarkers, were significantly associated with increased risk of cardiovascular disease occurrence and mortality. The possible cause was that elevated CRP levels were directly related to the occurrence of atherosclerosis and the risk of plaque rupture (42, 43). Besides, NLR was also one of the frequent inflammation biomarkers. With the increase in NLR, there was an increased risk of cardiovascular disease occurrence and death (44). Unlike traditional inflammation biomarkers, the ALI explored in this study was a novel inflammation biomarker that took into account both inflammation and nutritional status. It was well known that nutritional status was closely linked to stroke and cardiovascular disease. As previously mentioned, albumin and BMI have a remarkable impact on the mortality rate of stroke patients. The presence of the obesity paradox phenomenon rendered the ALI index not totally consistent with traditional inflammatory biomarkers in predicting the risk of cardiovascular disease death in stroke patients.

An intriguing finding from the study was that, when analyzing ALI as a categorical variable, the trend of CVD mortality risk for Quantile2 and Quantile3 groups did not show a significant decrease compared to Quantile1 group (*p*_trend_ = 0.13). However, when ALI was treated as a continuous variable within the multivariate-adjusted Cox model, a 9% reduction in CVD mortality risk for every 10 U increase in ALI. Figure 3B suggested a linear negative correlation between ALI levels and CVD mortality. This was consistent with the influence of every 10 U change in ALI on CVD mortality. One reason behind the observed inconsistency might be the differing statistical efficacies between categorical and continuous variables. Another reason could be that the sample size of this study was not sufficiently large. Moving forward, investigations should increase the sample size as a foundation to further evaluate the linkage between ALI and CVD mortality among stroke patients.

The current study has the following advantages. Firstly, the data used in this study originated from a large public database that was nationally representative, ensuring its credibility, reliability, and representativeness. Secondly, we employed a variety of statistical analysis methods to minimize interference from confounding factors on our findings, comprising the multivariable-adjusted Cox analysis, stratified analysis, and interaction analysis. Finally, ALI served as a comprehensive index that was both easy to derive and compute, proving very practical for clinical usage.

The current study has some limitations. On one hand, given that this was an observational research, it was not possible to definitively establish a causal link between ALI and the mortality associated with stroke patients. Future research with larger sample sizes and a prospective design was needed to clarify their causality. On the other hand, unknown confounding factors cannot be entirely eliminated, even with the use of various statistical methods to reduce bias.

## Conclusion

Briefly, our research determined for the first time that an elevation in ALI was closely linked to a decrease in the all-cause mortality risk of stroke patients. ALI and overall mortality exhibited an inverse J-shaped non-linear relationship, with an inflection point (lowest mortality risk) at 83.76. These results emphasized the importance of managing the ALI of stroke patients within a suitable range for their long-term survival (such as weight control, keep albumin in the normal range, and anti-inflammatory treatments). The dynamic changes in ALI were also beneficial for clinicians to establish personalized ALI standards to maximize the long-term survival of stroke patients.

## Data availability statement

The original contributions presented in the study are included in the article/Supplementary material, further inquiries can be directed to the corresponding author.

## Ethics statement

The studies involving humans were approved by the Institutional Review Board (or Ethics Committee) of the institutional review board of the National Center for Health Statistics, CDC (protocol #2005-06, #2011-17, #2018-01). The studies were conducted in accordance with the local legislation and institutional requirements. The participants provided their written informed consent to participate in this study.

## Author contributions

XC: Conceptualization, Methodology, Software, Writing – original draft. CH: Data curation, Formal analysis, Writing – original draft. ZG: Data curation, Formal analysis, Writing – original draft. HH: Data curation, Formal analysis, Writing – original draft. LY: Funding acquisition, Resources, Supervision, Validation, Writing – review & editing.
