# Supplementary material for: Association between advanced lung cancer inflammation index and all-cause and cardiovascular mortality among stroke patients: NHANES, 1999–2018
Source: Front Public Health. 2024 Apr 18;12:1370322. doi: 10.3389/fpubh.2024.1370322 (PMC11063327; doi:10.3389/fpubh.2024.1370322)
Supplement: Supplementary file 1 [file Table_1.DOC]

**Supplementary Online Content**

**Supplementary Methods**

**Supplementary Table S1.** Stratified analyses of the relationships of ALI with all-cause mortality in patients with stroke from the NHANES 1999–2018 cohort.

**Supplementary Table S2.** Stratified analyses of the relationships of ALI with CVD mortality in patients with stroke from the NHANES 1999–2018 cohort.

**Supplementary Table S3.** Stratified analyses of threshold effect about the relationships of ALI with all-cause mortality in patients with stroke from the NHANES 1999–2018 cohort.

**Supplementary Methods**

Races were categorized into White or Non-White. BMI was calculated by dividing weight in kilograms by height in meters squared. Regarding smoking status, it was grouped as never/former-smoker, or now-smoker, per their responses to whether they currently smoked and had spent at least 100 cigarettes in their life. Alcohol consumption was grouped by alcohol intake. The participants who consumed less than twelve drinks in a lifetime was categorized as a never drinker; those who consumed one to two drinks for female or one to two for male was categorized as a mild-moderate drinker; those who consumed more than two drinks for female and three drinks for male was categorized as a heavy drinker. Having a history of hypertension is grouped as taking anti-hypertensive medication for hypertension or a mean systolic blood pressure (SBP) greater than or equal to 140 mmHg and/or a mean diastolic blood pressure (DBP) greater than or equal to 90 mmHg when SBP and DBP were collected at MEC or self-reporting of doctor's diagnosis of hypertension. Having a history of diabetes was grouped as receiving oral hypoglycemic agents or insulin, the hemoglobin concentrations were higher than or equal to 6.5% or fasting glucose higher than or equal to 7.0 mmol/l, or random fasting glucose higher than or equal to 11.1 mmol/l or 2-hour glucose tolerance glucose higher than or equal to 11.1 mmol/l or self-reported of a doctor's diagnosis of diabetes.

**Supplementary Table S1. Stratified analyses of the relationships of ALI with all-cause mortality in patients with stroke from the NHANES 1999–2018 cohort**

| **Characteristics** | **ALI** |  |  |  |  |
| --- | --- | --- | --- | --- | --- |
|  | **Quantile 1**  32.67 [2.89,44.11] | **Quantile 2**  54.69  (44.11,68.18] | **Quantile 3**  88.94  (68.18,893.63] | ***P* for trend** | ***P* for interaction** |
| Participants, n | 480 | 480 | 480 |  |  |
| Age |  |  |  |  | 0.09 |
| ≤60 | ref | 0.58(0.25,1.34) | 0.63(0.30,1.33) | 0.78 |  |
| >60 | ref | 0.69(0.54,0.88) | 0.42(0.33,0.53) | 0.004 |  |
| Gender |  |  |  |  | 0.36 |
| Female | ref | 0.54(0.38,0.76) | 0.40(0.29,0.56) | 0.06 |  |
| Male | ref | 0.78(0.59,1.04) | 0.55(0.39,0.76) | <0.001 |  |
| Race |  |  |  |  | 0.24 |
| White | ref | 0.64(0.49,0.83) | 0.44(0.33,0.58) | <0.0001 |  |
| Non-White | ref | 0.80(0.47,1.35) | 0.61(0.40,0.94) | 0.02 |  |
| Smoke status |  |  |  |  | 0.84 |
| No/Former | ref | 0.65(0.50,0.84) | 0.46(0.36,0.60) | 0.002 |  |
| Now | ref | 0.69(0.41,1.14) | 0.59(0.34,1.00) | 0.68 |  |
| Hypertension |  |  |  |  | 0.18 |
| No | ref | 0.43(0.25,0.74) | 0.55(0.29,1.01) | 0.39 |  |
| Yes | ref | 0.71(0.54,0.93) | 0.45(0.36,0.57) | 0.02 |  |
| Diabetes |  |  |  |  | 0.73 |
| No | ref | 0.71(0.54,0.94) | 0.53(0.38,0.74) | 0.13 |  |
| Yes | ref | 0.58(0.39,0.86) | 0.35(0.24,0.53) | 0.04 |  |
| Total cholesterol |  |  |  |  | 0.35 |
| ≤5.18 | ref | 0.78(0.60,1.03) | 0.54(0.40,0.74) | <0.0001 |  |
| >5.18 | ref | 0.53(0.35,0.80) | 0.39(0.27,0.56) | <0.0001 |  |
| Uric acid |  |  |  |  | 0.45 |
| ≤420 | ref | 0.68(0.52,0.89) | 0.52(0.40,0.66) | <0.0001 |  |
| >420 | ref | 0.57(0.37,0.88) | 0.32(0.18,0.57) | <0.001 |  |
| LDH |  |  |  |  | 0.12 |
| <1 | ref | 1.03(0.62,1.70) | 0.77(0.43,1.38) | 0.37 |  |
| ≥1 | ref | 0.60(0.46,0.78) | 0.43(0.33,0.56) | <0.0001 |  |

**Supplementary Table S2. Stratified analyses of the relationships of ALI with CVD mortality in patients with stroke from the NHANES 1999–2018 cohort**

| **Characteristics** | **ALI** |  |  |  |  |
| --- | --- | --- | --- | --- | --- |
|  | **Quantile 1**  32.67 [2.89,44.11] | **Quantile 2**  54.69  (44.11,68.18] | **Quantile 3**  88.94  (68.18,893.63] | ***P* for trend** | ***P* for interaction** |
| Participants, n | 480 | 480 | 480 |  |  |
| Age |  |  |  |  | 0.30 |
| ≤60 | ref | 0.87(0.26,2.87) | 0.63(0.22,1.82) | 0.54 |  |
| >60 | ref | 0.68(0.48,0.97) | 0.37(0.24,0.57) | 0.05 |  |
| Gender |  |  |  |  | 0.20 |
| Female | ref | 0.49(0.31,0.79) | 0.40(0.23,0.70) | 0.42 |  |
| Male | ref | 0.93(0.56,1.54) | 0.39(0.22,0.68) | 0.001 |  |
| Race |  |  |  |  | 0.41 |
| White | ref | 0.64(0.43,0.96) | 0.39(0.24,0.63) | <0.0001 |  |
| Non-White | ref | 0.89(0.46,1.72) | 0.58(0.32,1.04) | 0.05 |  |
| Smoke status |  |  |  |  | 0.93 |
| No/Former | ref | 0.78(0.53,1.15) | 0.48(0.31,0.74) | 0.28 |  |
| Now | ref | 0.44(0.20,0.97) | 0.37(0.18,0.76) | 0.82 |  |
| Hypertension |  |  |  |  | 0.85 |
| No | ref | 0.43(0.18,1.00) | 0.30(0.10,0.90) | 0.59 |  |
| Yes | ref | 0.74(0.50,1.09) | 0.47(0.31,0.72) | 0.16 |  |
| Diabetes |  |  |  |  | 0.39 |
| No | ref | 0.75(0.49,1.15) | 0.37(0.22,0.64) | 0.02 |  |
| Yes | ref | 0.61(0.34,1.10) | 0.49(0.27,0.89) | 0.2 |  |
| Total cholesterol |  |  |  |  | 1.00 |
| ≤5.18 | ref | 0.73(0.47,1.14) | 0.47(0.29,0.75) | 0.001 |  |
| >5.18 | ref | 0.62(0.36,1.09) | 0.35(0.20,0.64) | <0.001 |  |
| Uric acid |  |  |  |  | 0.46 |
| ≤420 | ref | 0.65(0.44,0.96) | 0.47(0.30,0.73) | <0.001 |  |
| >420 | ref | 0.75(0.39,1.44) | 0.29(0.13,0.67) | 0.002 |  |
| LDH |  |  |  |  | 0.24 |
| <1 | ref | 1.23(0.64,2.37) | 0.59(0.24,1.47) | 0.24 |  |
| ≥1 | ref | 0.56(0.37,0.82) | 0.38(0.25,0.58) | <0.0001 |  |

**Supplementary Table S3. Stratified analyses of threshold effect about the relationships of ALI with all-cause mortality in patients with stroke from the NHANES 1999–2018 cohort**

| **Characteristics** | **ALI** | | | |
| --- | --- | --- | --- | --- |
|  | <83.76 | >83.76 | ***P* for trend** | ***P* for interaction** |
| Age |  |  |  | 0.14 |
| ≤60 | ref | 0.83(0.46,1.51) | 0.54 |  |
| >60 | ref | 0.60(0.46,0.78) | <0.001 |  |
| Gender |  |  |  | 0.42 |
| Female | ref | 0.71(0.50,1.01) | 0.06 |  |
| Male | ref | 0.59(0.40,0.88) | 0.01 |  |
| Race |  |  |  | 0.34 |
| White | ref | 0.63(0.45,0.89) | 0.01 |  |
| Non-White | ref | 0.81(0.55,1.18) | 0.27 |  |
| Smoke status |  |  |  | 0.82 |
| No/Former | ref | 0.66(0.50,0.87) | 0.003 |  |
| Now | ref | 0.70(0.39,1.26) | 0.23 |  |
| Hypertension |  |  |  | 0.18 |
| No | ref | 1.01(0.51,1.98) | 0.98 |  |
| Yes | ref | 0.64(0.49,0.83) | <0.001 |  |
| Diabetes |  |  |  | 0.34 |
| No | ref | 0.60(0.39,0.93) | 0.02 |  |
| Yes | ref | 0.74(0.54,0.99) | 0.05 |  |
| Total cholesterol |  |  |  | 0.72 |
| ≤5.18 | ref | 0.75(0.51,1.12) | 0.16 |  |
| >5.18 | ref | 0.60(0.40,0.90) | 0.01 |  |
| Uric acid |  |  |  | 0.42 |
| ≤420 | ref | 0.71(0.53,0.95) | 0.02 |  |
| >420 | ref | 0.56(0.32,0.98) | 0.04 |  |
| LDH |  |  |  | 0.42 |
| <1 | ref | 0.58(0.30,1.12) | 0.1 |  |
| ≥1 | ref | 0.70(0.52,0.94) | 0.02 |  |
